# Structural modification of isomorphous SO_4_^2−^-doped K_2_FeO_4_ for remediating the stability and enhancing the discharge of super-iron battery

**DOI:** 10.1098/rsos.180919

**Published:** 2019-01-23

**Authors:** Chao Yan, Lingyue Zhu, Jing Dong, Di Gu, Hong Jiang, Baohui Wang

**Affiliations:** School of Chemistry and Chemical Engineering, Northeast Petroleum University, No. 199 Fazhan Road, High-tech Development Zone, Daqing 163318, People's Republic of China

**Keywords:** ferrates, K_2_FeO_4_, super-iron battery, discharge, capacity, stability

## Abstract

In the paper, the isomorphous $SO_{4}^{2-}$ doped K_2_FeO_4_, aimed at the remediation of the discharge and stability of the super-iron battery, was first synthesized for doping and reforming the K_2_FeO_4_ crystalline structure via a facile co-precipitation and mechanochemistry. Afterwards, the compared cathodes were assembled by the undoped and doped K_2_FeO_4_ for an evaluation of the discharge and stability in the AAA super-iron battery system. The results show that the small amounts of K_2_SO_4_ were doped into the K_2_FeO_4_ in the calculated form of K_2_Fe_1−x_S_x_O_4_ by the isomorphous substitution. The doped K_2_FeO_4_ cathodes/batteries exhibited an excellent discharge with a normal discharge profile. The cathodes doped by two techniques had significantly enhanced the discharge capacity of the super-iron battery with an increase of 10–30% compared to the undoped K_2_FeO_4_. Moreover, the stability of the K_2_FeO_4_ cathodes was obviously remediated by the isomorphous $SO_{4}^{2-}$ doping. The shelf time of the doped K_2_FeO_4_ cathodes was prolonged by an increase of about 10% in comparison of the undoped K_2_FeO_4_ cathode. The desirable enhancements could be attributed to doping and reforming the similar building block and isomorphous $SO_{4}^{2-}$ into the $\mathrm{Fe}O_{4}^{2-}$ tetrahedral and crystalline in the form of the isomorphous substitution and filling vacancies.

## Highlights

1. The inherent instability of the K_2_FeO_4_ was attributed to the structural defects and vacancies.
2. The isomorphous $SO_{4}^{2-}$ was first doped for the reforming of the K_2_FeO_4_ crystalline.
3. The doped K_2_FeO_4_ super-iron batteries significantly exhibited an excellent discharge and stability.
4. The enhancements are attributed to the doping and reforming of the similar size and isomorphous $SO_{4}^{2-}$ to the $\mathrm{Fe}O_{4}^{2-}$ tetrahedral.

## 1. Introduction

Ferrate (VI) compounds have been extensively studied since they were initially discovered a century ago, as recorded by the huge amounts of publications on subjects of water treatment [1–7], super-iron battery [8–10], organic synthesis [11] and photocatalytic oxidation [12]. The ferrate chemistry has been revealed and kept relatively clear in the preparation, structure, properties and redox characteristics as an active oxidizing agent [1,13,14]. Owing to the highly oxidized valence, multiple electron transfers and high intrinsic energy, we introduced a green battery with high energy, environmental benignity and low cost in 1999 [14], which adopted the ferrate (VI) compounds as the cathode materials, referred to as a super-iron battery. Afterwards, the positive advantages of the super-iron battery have been attracting a growing research attention. Among these Fe (VI) cathodes, K_2_FeO_4_ has been paid the most emphasis to because of its high intrinsic capacity (406 mA h g^−1^) and appropriate solid-state stability [15,16].

Ferrate ion has the molecular formula, $\mathrm{Fe}O_{4}^{2-}$, and is a very strong oxidant in the aqueous system when ferrate (VI) compounds are dissolved in water. Because the redox potential of the ferrate ion (2.20 V, standard hydrogen electrode (SHE)) is greater than H_2_O under acidic conditions, oxygen is evolved and ferric hydroxide is precipitated.

1.1 $$4K_{2}\mathrm{Fe}O_{4}+10H_{2}O=4\mathrm{Fe}{\left(\mathrm{OH}\right)}_{3}+8\mathrm{KOH}+3O_{2}↑.$$

It was supposed that the ferrates are unstable in the aqueous solution. So, the inherent thermodynamic instability of $\mathrm{Fe}O_{4}^{2-}$ in water has restrictions for its wide applications.

In the super-iron battery system, some researchers have demonstrated that the initially unstable/defective crystal structure and the later formation of Fe(OH)_3_ coated layer on the K_2_FeO_4_ are likely to degrade the discharge and stability of K_2_FeO_4_-based super-iron battery [15,17]. Therefore, the practical discharge capacity of K_2_FeO_4_ has been presented by much less than the theoretical one of 406 mA h g^−1^ in these reports. Moreover, it was obviously decreased with an increase of the shelf time during the storage [18–21]. Summarily, the two defects have been attributed to the inactive and unstable K_2_FeO_4_ cathode in the present investigation [22,23]. Thereby, these fateful disadvantages restrict the large-scale application and development of the K_2_FeO_4_ super-iron battery. It is necessary to explore a new technique to enhance the practical capacity and stability of K_2_FeO_4_ cathode.

Recently, many attempts have been intensively implemented for the improvements of the instability of the ferrate (VI) compounds and super-iron battery.

For the stability of the ferrate solution, more works have been presented to study the effect of the coexisting ions and buffers on the stabilization in the aqueous solution [24]. The decomposition rate of the ferrate solutions depends strongly on the initial ferrate concentration, coexisting ions, pH and temperature of the solution [5,14,25]. Our current investigation displayed that the adoption of KIO_4_ equalizer greatly increased the lifetime of $\mathrm{Fe}O_{4}^{2-}$ in water by orders of magnitude. The stabilization mechanism was supposed to occur in the effect of the redox equilibrium of the $\mathrm{Fe}O_{4}^{2-}$ and the ${\mathrm{IO}}_{4}^{-}$ species, as well as the formation of an oxidizing chemical environment [26].

To enhance the charge transfer and stability of the ferrate cathodes, many types of inorganic and organic compounds were coated on the ferrates for a modification. Some ceramics materials such as ZrO_2_ and yttria were coated on K_2_FeO_4_ by Licht *et al.* [15,19] and Zhang *et al.* [27] for enhancing the conductivity and stability of the K_2_FeO_4_ battery. Walz reported that BaFeO_4_ was coated with nanoparticulate thin films of TiO_2_ and SiO_2_, which was prepared by sol-gel techniques [17,19]. Yang revealed that organic compounds of (2,3-Naphthalocyanine, tetra- phenyl porphyrin, phthalocyanine) were employed as coatings to enhance the stability of K_2_FeO_4_ [9,23,28]. The protective coatings enabled the separation of the electrolyte and K_2_FeO_4_ for a reduction of destroying the K_2_FeO_4_ with a relative lift of the charge transfer ability. However, the mixed compounds still indicated a comparative conduct with a low stability [23,24,27,29–31]. For the improvement, poly(3-hexylthiophene)-coated K_2_FeO_4_ was prepared to enhance the capacity and stability of K_2_FeO_4_. These positive effects were considered to the *in situ* formation of a two-layer film on the surface of the K_2_FeO_4_ crystal, which keeps the electrolyte from direct contact with K_2_FeO_4_ and reduces the resistance of charge transfer. Huang *et al.* [24] conducted the coating of potassium ferrate (VI) by phthalocyanine (H_2_Pc), which showed that the decomposition of K_2_FeO_4_ in the electrolyte was obviously suppressed by H_2_Pc coating with a short immersion time, and the capacity of the electrode was enhanced in some sense. The latest progress reported the addition of plastically bonded cathode by using non-stoichiometric binary titanium oxides and Magnèli phases for improving the performance of the ferrate cathode [32]. Despite the potential superior improvements to the insulation and conductivity by the various coatings, the development of super-iron batteries has been slowed down due to some intrinsic limitations facing their chemical instability, inactive materials and high self-discharge. Therefore, the research focuses have to be switched to the inherent structure of ferrate (VI) compounds for the activation and stabilization via the modification and doping of the crystalline structure.

The crystal structure of K_2_FeO_4_ was revealed by Hoope *et al.* in 1982 [33]. K_2_FeO_4_ has orthorhombic crystalline and space group Pnam. The K_2_FeO_4_ compound is isomorphous with K_2_SO_4._ The tetrahedral $\mathrm{Fe}O_{4}^{2-}$ has three independent Fe-O bond lengths 164.5, 165.3 and 165.6 pm, respectively. These are slightly longer than those in the isomorphous K_2_SO_4_.

**Figure 1.**
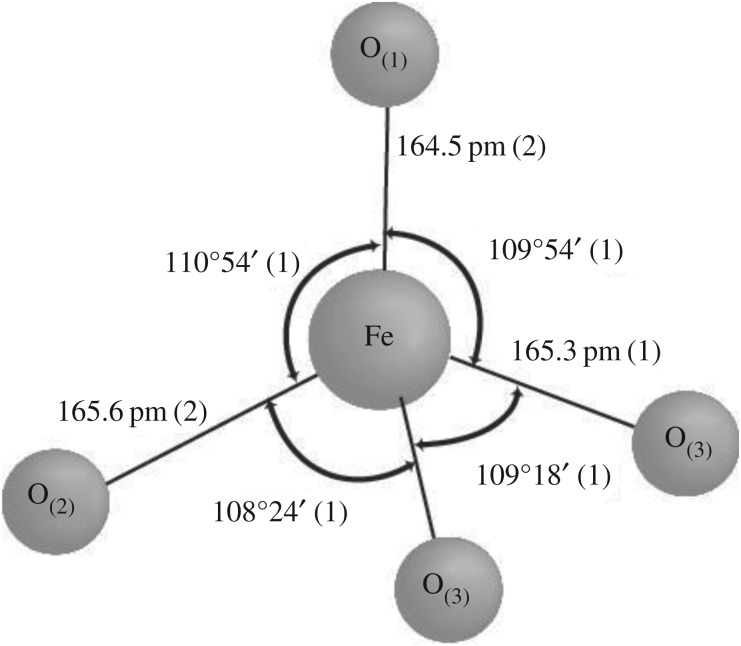
The $\mathrm{Fe}O_{4}^{2-}$ anion, Fe-O bond distances and angles.

The ferrate ion has a tetrahedral structure for a building block as shown in figure 1. The bond angles are close to the 109.5° required for a perfect tetrahedron, but with slight differences. The environments of the two K atoms were calculated to a distance of 350 pm. The coordination number for one is ten with contacts to O between 272.5 and 326.0 pm. The coordination for the other is nine with contacts 273.2–314.5 pm. The Fe–O bond distances are slightly longer than the S–O distances (149 pm).

Based on the data of the bond distances, the $\mathrm{Fe}O_{4}^{2-}$ tetrahedral has different bond lengths. It was supposed that the tetrahedron has a deformed structure with tension for basically showing a trend of an unstable structure. The unstable trend originated from the basic unit cell can be expected that crystal is not stable. On the other hand, the crystal defect should exist in the structure because of the cell vacancy or overfill, which is also an unstable factor. To make sense, we can find the equivalent atom, unit cell and isomorphous crystalline with the $\mathrm{Fe}O_{4}^{2-}$ tetrahedral to dope the appropriate vacancies and replace Fe-alternative sites for filling the in/on-crystal defects and forming the isomorphous substitution of K_2_Fe_1−x_S_x_O_4_ complex salt. The alternative strategy would be supposed to effective for improving the activation and stability of K_2_FeO_4_ via the doping of the tetrahedron unit and crystalline. The doping of the in/out-crystal and vacancies is intended for the stability, and the Fe substitution is for the activation.

**Figure 2.**
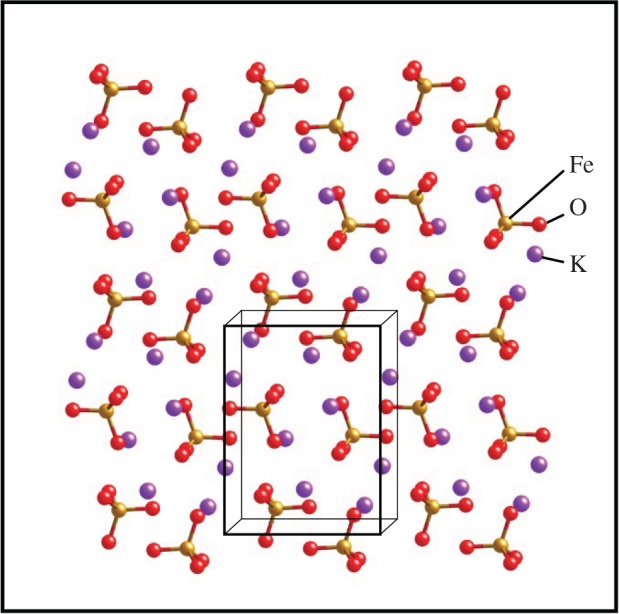
The orthorhombic crystalline of K_2_FeO_4_.

**Figure 3.**
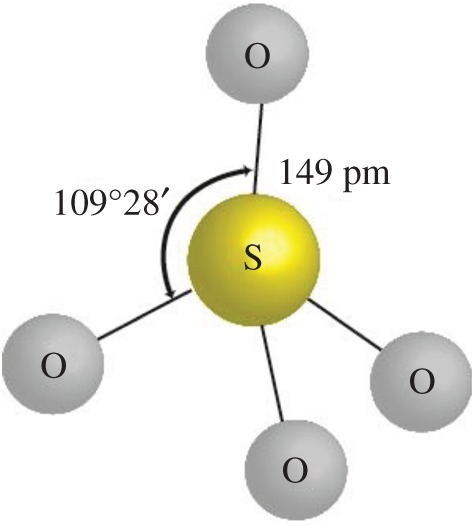
The $SO_{4}^{2-}$ anion; Fe-O bond distances and angles.

According to our knowledge, $SO_{4}^{2-}$ has isomorphous crystalline such as the $\mathrm{Fe}O_{4}^{2-}$ tetrahedral building block with slightly similar bond length and radical size as shown in figure 2. It can be expected that $SO_{4}^{2-}$ is a suitable candidate for the doping with an easy coupling (figure 3).

In this paper, an alternative strategy for remediating the discharge and stability of super-iron battery was conducted by the structural modification of isomorphous $SO_{4}^{2-}$ doped K_2_FeO_4_. The isomorphous $SO_{4}^{2-}$ doped K_2_FeO_4_ was first performed by a double-ions co-precipitation (chemical doping) and mechanochemistry (mechanical doping). Afterwards, the cathodes were prepared by the undoped and doped K_2_FeO_4_ for the battery. The AAA super-iron batteries were installed for an evaluation of the discharge and stability in the battery system.

## 2. Experimental

### 2.1. Preparation of K_2_FeO_4_

K_2_FeO_4_ can be synthesized with four synthetic routes. These are: (i) high-temperature dry oxidation, (ii) electrochemical method, (iii) wet chemical oxidation of iron, and (iv) using chemical oxidizing agents.

In the paper, the K_2_FeO_4_ was chemically synthesized by the oxidation of ferric salts in alkaline hydroxide media by using analytical grade reagents. The preparation of the K_2_FeO_4_ has been described in detail in our previous papers [8–10,14,16]. Briefly, the wet chemical oxidation method includes the oxidation of ferric ion by an alkaline potassium hypochlorite solution (preferably with high purity, i.e. more than 12%) as an effective oxidizing reagent in the presence of potassium hydroxide which may yield a high concentration of the potassium ferrate (VI). The reaction involved in the preparation process is given as

2.1 $$\mathrm{Fe}{\left(NO_{3}\right)}_{3}\cdot 9H_{2}O+3/2\mathrm{KClO}+5\mathrm{KOH}=K_{2}\mathrm{Fe}O_{4}↓+3/2\mathrm{KCl}+3\mathrm{KN}O_{3}+23/2H_{2}O.$$

This procedure produced a high yield of potassium ferrate (VI). Many separation steps were followed by several purifications of the filtration, recrystallization, washing and drying processes. The preparation yielded 97–99% purity of K_2_FeO_4_.

### 2.2. Analysis of purity and stability of K_2_FeO_4_

The K_2_FeO_4_ purity was determined by redissolution and oxidation of chromite (chromite method) [17] in which the chromate generated was titrated with a standard ferrous ammonium sulfate solution, by using a sodium diphenylamine sulfonate indicator. In brief, the pure K_2_FeO_4_ or samples taken from the battery were dissolved into a solution. The solutions plus the indicator were titrated by a standard solution in the titration to a colour change from purple to green. Then, the K_2_FeO_4_ purity was determined by the normality.

The chromite method was used to determine the stability of K_2_FeO_4_ when the oxidizing capacity of samples was measured over time and compared to the equivalents (three electrons) of Fe (VI → III) within the compounds.

### 2.3. Doping of K_2_FeO_4_ by isomorphous $SO_{4}^{2-}$

The doping was conducted by employing the double-ions co-precipitation (chemical doping) and mechanochemistry (mechanical doping).

The double-ions co-precipitation was started by the recrystallization of the K_2_FeO_4_. Tens grams of the pure K_2_FeO_4_ were dissolved in 2.57 M KOH, and quickly filtered through a funnel with two layers of the glass filter paper of 230 mm diameter, then, a 0.5–5% K_2_SO_4_/2.57 M KOH solution was directly added to the above solution, and totally into 0°C, 12 M KOH. The solution was stirred for 15 min at 3°C, then the solution was filtered onto a glass filter. The precipitate of the K_2_SO_4_ doped K_2_FeO_4_ was successively rinsed four times with *n*-hexane, four times with methanol and finally three times with diethyl ether. The K_2_FeO_4_ was dried for 30–60 min at room temperature under vacuum. The product was sealed in a vial for a test use of the stability and discharge.

The mechanochemical doping is a facile solid–solid chemical synthesis by a mechanical force of the grinding. The mechanochemical synthesis of BaFeO_4_ was succeeded by a grinding of the solid ferrate plus solid alkaline for an exchange of metal sites in our past work [34]. The procedure is described for a manually grinding doping as follows: taking tens grams of the pure K_2_FeO_4_ plus the 1–2% K_2_SO_4_ to an agate mortar, the mixture was ground for 30 min by the manual mode. The product was kept in a sealed vial for ready use.

The per cent of the doped $SO_{4}^{2-}$ contents were determined by a chemical method of dissolving the sample and an instrumental analysis of an ion chromatography. The sample of tens milligrams was dissolved by 2 M hydrochloric acid solution until a complete decomposition of K_2_FeO_4_. Then, the solution was filtered to collect the $SO_{4}^{2-}$ effluent. The effluent was appropriately diluted for the quantitative $SO_{4}^{2-}$ analysis of the ion chromatography (IC, Shimadzu, Japan).

### 2.4. X-ray diffractometer and scanning electron microscope measurements

The crystal structures were characterized by an X-ray diffractometer (XRD, Rigaku D/MAX-2200) with Cu K*α* source in the range of 2*θ* = 10–80°.

The morphology of the undoped and doped K_2_FeO_4_ structures was determined by a field-emission scanning electron microscope (FESEM, Zeiss SigmaHV).

### 2.5. Fabrication and discharge of AAA super-iron batteries by the undoped and doped K_2_FeO_4_

The experimental details were presented elsewhere for fabrication and discharge of AAA super-iron batteries [14].

A composite cathode was formed by mixing a specified mass of the undoped or doped K_2_FeO_4_ with an indicated weight per cent of fine graphite. In the experiments, the cell components, including the case, separator, collector and Zn paste anode, were used from standard commercial AAA alkaline cells (a cylindrical cell configuration with diameter 10.1 mm and a 42 mm case height). The cathode mix contained the 75% K_2_FeO_4_ + 10% graphite (47 µm) + 15% 13.5 M KOH electrolyte in the total mass of 4.8 g (active constituent 3.6 g). The excess Zn-gel paste was used as an anode. The K_2_FeO_4_ mix with the electrolyte of saturated KOH was pressed into proper rings, followed by insertion of the ring, separator, Zn anode mix, gasket, and anode collector and sealing of the cell. The cathode composites contain various cathodes fabricated by the undoped and doped K_2_FeO_4_ with the same amounts of graphite. Cells were discharged at a constant resistance load (220 Ω). Cell potential variation over time was recorded via a data acquisition on a PC, and cumulative discharge, as ampere-hours, determined by the subsequent integration. The theoretical discharge capacity is calculated by the (three Faraday per mole, converted to ampere-hours) measured cathode mass of the Fe (VI) salt. The three electron Fe (VI) faradaic efficiency is determined by comparison of the measured cumulative ampere-hours of discharge to the theoretical discharge capacity.

For a measurement of the stability, a part of the cathode mix was left in a sealed vial for a time-interval test. The residual content of the undoped or doped K_2_FeO_4_ was determined by a chemical analysis mentioned in the above section.

## 3. Results and discussion

### 3.1. Analysis of composition and formula of the $SO_{4}^{2-}$-doped K_2_FeO_4_

The doped samples were analysed for the determination of contents of the K_2_FeO_4_ and K_2_SO_4_ by the above-mentioned methods. The formulae of complex salts were calculated by forming the isomorphous substitution of K_2_Fe_1−x_S_x_O_4_ complex based on the weight percentages of the K_2_FeO_4_ and K_2_SO_4_ (two building blocks).

The analytical data are listed in tables 1 and 2.

**Table 1.** Analytical and calculation data of the $SO_{4}^{2-}$ doped K_2_FeO_4_ by the double-ions co-precipitation. Initial K_2_FeO_4_ content: 97%.

adding percentage of K_2_SO_4_ (%)	0.5	1.0	5.0
practical percentage of K_2_SO_4_ (w/w, %) (IC)	0.1	0.5	2.0
K_2_FeO_4_ contents (w/w, %) (chromite)	96	95	94
salt formula (K_2_Fe_1−x_S_x_O_4_)	K_2_Fe_0.998_S_0.002_O_4_	K_2_Fe_0.994_S_0.006_O_4_	K_2_Fe_0.976_S_0.024_O_4_

**Table 2.** Analytical and calculation data of the $SO_{4}^{2-}$ doped K_2_FeO_4_ by the mechanochemistry. Initial K_2_FeO_4_ content: 97%.

adding percentage of K_2_SO_4_ (%)	0.5	1.0	2.0
practical percentage of K_2_SO_4_ (w/w, %) (IC)	0.5	1.0	2.0
K_2_FeO_4_ contents (w/w, %) (chromite)	96	95	94
salt formula (K_2_Fe_1−x_S_x_O_4_)	K_2_Fe_0.994_S_0.006_O_4_	K_2_Fe_0.988_S_0.012_O_4_	K_2_Fe_0.976_S_0.024_O_4_

The data and formulae show that the small amounts of K_2_SO_4_ were obviously doped into the K_2_FeO_4_ in the form of K_2_Fe_1−x_S_x_O_4_ by the isomorphous substitution. It is expected that the capacity of the active materials could be significantly reduced by the doping of the inactive additives.

**Figure 4.**
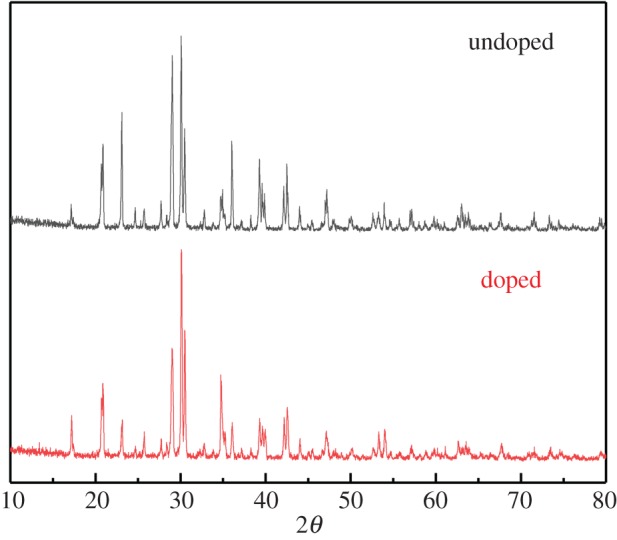
The XRD patterns of the undoped and doped K_2_FeO_4_ samples.

The XRD patterns of the undoped and doped K_2_FeO_4_ samples are shown in figure 4.

Both XRD patterns demonstrated no significant differences in both materials. The doped XRD pattern seems to slightly improve the crystalline. Obviously, new $SO_{4}^{2-}$ peaks were not found in the pattern of the doped K_2_FeO_4_ materials compared to the pure K_2_FeO_4_ because of the same pattern of two crystalline and the small existing amounts of $SO_{4}^{2-}$.

**Figure 5.**
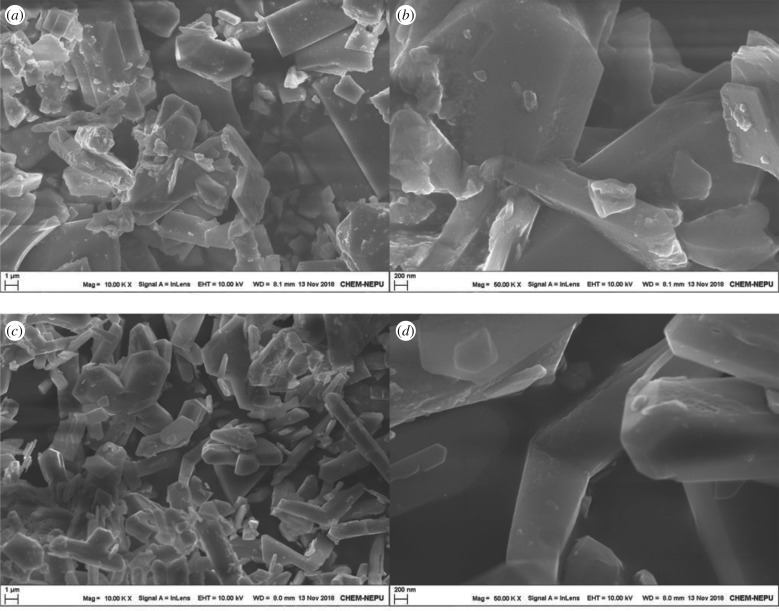
The SEM images of the undoped and doped K_2_FeO_4_ samples ((*a*) the undoped sample in 1 µm scale, (*b*) the undoped sample in 200 nm scale, (*c*) the doped sample in 1 µm scale and (*d*) the doped sample in 200 nm scale).

The SEM images of the undoped and doped K_2_FeO_4_ samples are shown in figure 5.

The doped image demonstrates the existence of the well-ordered and distributed crystalline compared to the undoped one. The crystal size can be scaled in the SEM images.

### 3.2. Discharge of the $SO_{4}^{2-}$-doped K_2_FeO_4_ cathodes

For the evaluation of the cathode discharge, we assembled the AAA-type alkaline super-iron batteries by using the as-synthesized materials.

**Figure 6.**
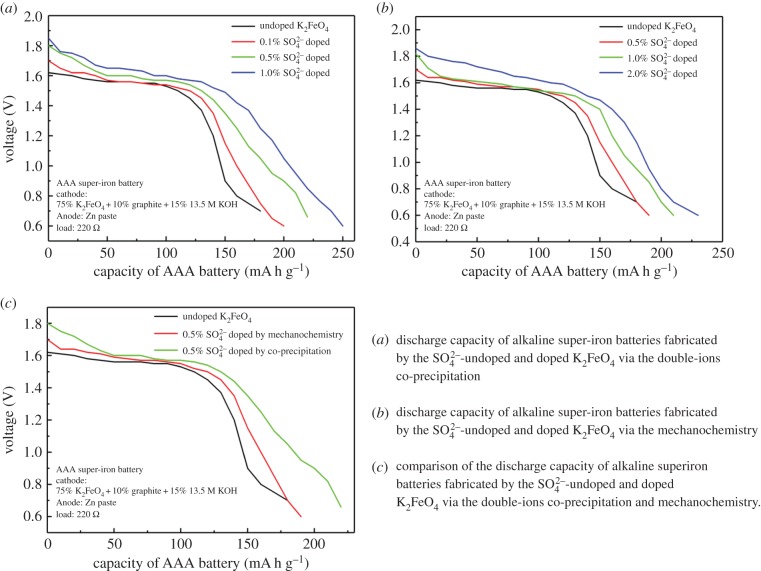
The discharge analysis of the $SO_{4}^{2-}$-doped K_2_FeO_4_ cathodes compared to the undoped K_2_FeO_4_.

The discharge curves of the $SO_{4}^{2-}$-undoped and doped K_2_FeO_4_, by using the double-ions co-precipitation and mechanochemistry, are shown in figure 6*a*,*b*. Both the undoped and doped K_2_FeO_4_ cathodes/batteries displayed excellent discharge characteristics with similar discharge profiles. The two doping techniques had significantly enhanced the discharge capacity of the K_2_FeO_4_ super-iron battery.

For the synthesis of the co-precipitation, the capacity was lifted with an increase of the doping amounts in the given range. Even with a small percentage of the doping, the capacity was increased to some extent. The high capacity was reached to 225 mA h g^−1^ in the 1.0% doping, which is higher than 160 mA h g^−1^ of the undoped K_2_FeO_4_.

Even though using the synthesis of the limitedly effective mechanochemistry, the capacity was promoted with an increase of the doping amounts. Even with a small percentage of the doping, the capacity was increased to some extent.

This enhancement could be attributed to the following reform: the similar size and isomorphous $SO_{4}^{2-}$ could be doped into the $\mathrm{Fe}O_{4}^{2-}$ tetrahedral and crystalline to produce the isomorphous substitution of the K_2_Fe_1−x_S_x_O_4_ complex. As a result, the crystal structure (bond length and angle) and environments were altered with a reformation and redistribution. The effective activation ensures the improvement of the ferrate cathodes by lifting the coulombic force and conduction.

The comparative results in figure 6*c* indicate that the doping method affected the discharge capacity of alkaline super-iron batteries fabricated by the $SO_{4}^{2-}$-doped K_2_FeO_4_. The double-ions co-precipitation presented the high capacity of the cathode in comparison to the mechanochemistry.

### 3.3. Stability of the $SO_{4}^{2-}$-doped K_2_FeO_4_ cathode

In order to investigate the stability of the $SO_{4}^{2-}$-doped K_2_FeO_4_ cathode, the same compositions of the cathode as the AAA super-iron batteries (75% K_2_FeO_4_ + 10% graphite and 15% 13.5 M KOH) were prepared in the sealed vial at the room temperature. The sampling and test were conducted by the time interval.

**Figure 7.**
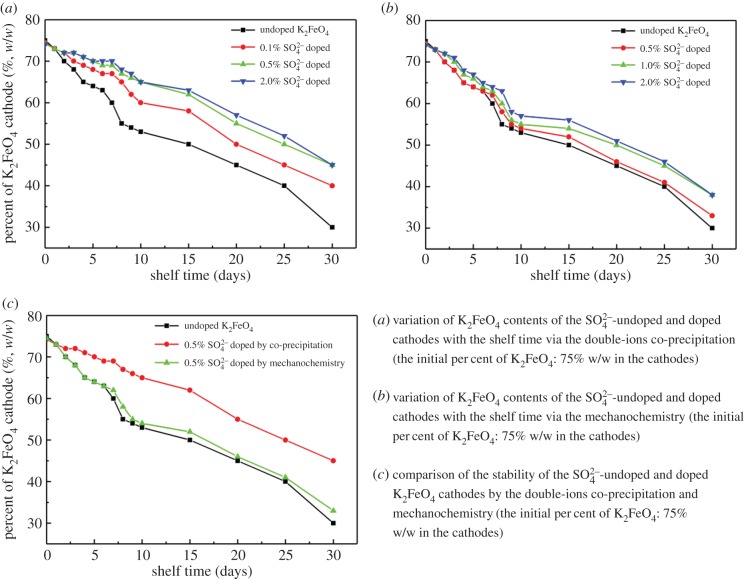
The stability analysis of the $SO_{4}^{2-}$-doped K_2_FeO_4_ cathodes compared to the undoped K_2_FeO_4_.

In the course of the tracking analysis, the results are exhibited in figure 7*a* (by the double-ions co-precipitation) and figure 7*b* (by the mechanochemistry). It can be seen that both doped K_2_FeO_4_ cathodes demonstrated an excellent stability via two doping techniques. The doped $SO_{4}^{2-}$ materials had evidently improved the stability of the K_2_FeO_4_ cathode.

For the synthesis of the co-precipitation, the stability was boosted with an increase of the doping amounts. In comparison, the life of the cathodes by the double-ions co-precipitation, to some extent, was greater than the one by the mechanochemistry as shown in figure 7*c*. This result was consistent with the discharge capacity of the cathodes.

The features could be explained by doping the similar $SO_{4}^{2-}$ into the in/out-crystalline and vacancies: the deformed structure and vacancies, and filling and reforming the sites for enhanced the stability.

### 3.4. Kinetics of the stability of the $SO_{4}^{2-}$-doped K_2_FeO_4_ cathodes

The detailed investigation of Fe (VI) stability is critical when its potential use for the battery in the aqueous alkaline medium is considered. It is known that ferrates (VI) are unstable in an aqueous medium and the extemporaneous decay of ferrates (VI) in water produces molecular oxygen and iron hydroxide.

The decomposition of the K_2_FeO_4_ cathode in aqueous KOH solution follows by equation (3.1)

3.1 $$4K_{2}\mathrm{Fe}O_{4}+10H_{2}O=4\mathrm{Fe}{\left(\mathrm{OH}\right)}_{3}+8\mathrm{KOH}+3O_{2}↑.$$

The rate equation can be simplified as equation (3.2)

3.2 $$\frac{-d[K_{2}{\mathrm{FeO}}_{4}]}{dt}=k{\left[K_{2}\mathrm{Fe}O_{4}\right]}^{n},$$

where −d[K_2_FeO_4_]/d*t* is the reaction rate, *t* is the reaction time, *k* is the kinetic constant of this reaction and *n* is the order of this reaction.

For the composition of K_2_FeO_4_, generally, it follows the one-order reaction.

The equation is given by

3.3 $$\frac{-d[K_{2}\mathrm{Fe}O_{4}]}{dt}=kt.$$

**Figure 8.**
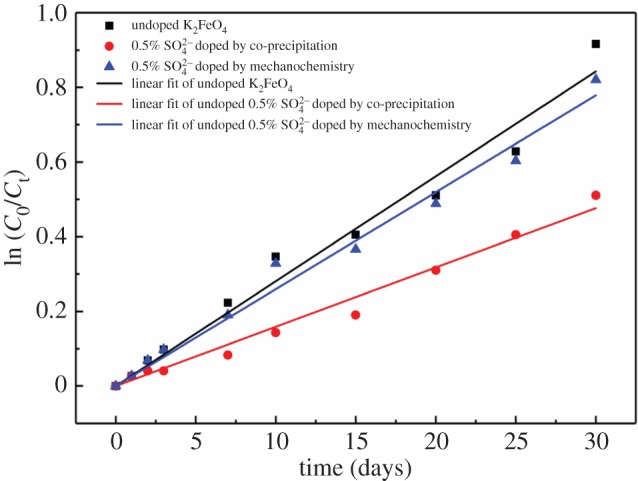
Kinetics of the decomposition of the $SO_{4}^{2-}$-undoped and doped K_2_FeO_4_ cathodes by the double-ions co-precipitation and mechanochemistry (the initial percentage of K_2_FeO_4_: *C*_0_ = 75% w/w in the cathodes).

Based on the data shown in figure 7*c*, the curves of the kinetics are plotted in figure 8.

The results demonstrated that the one-order kinetics is represented for the decomposition of the $SO_{4}^{2-}$-undoped and doped K_2_FeO_4_ cathodes with a good fitting degree. The kinetics data are summarized in table 3.

**Table 3.** Summary of the kinetic equation of the stability.

doping type	kinetic equation	*k*	correlation degree (*R*^2^)
Undoped K_2_FeO_4_	ln(*C*_0_/*C*_t_) = 0.0281t	0.0281	0.98848
0.5% $SO_{4}^{2-}$ doped by co-precipitation	ln(*C*_0_/*C*_t_) = 0.0159t	0.0159	0.99081
0.5% $SO_{4}^{2-}$ doped by mechanochemistry	ln(*C*_0_/*C*_t_) = 0.02597t	0.0260	0.99237

From table 3, *k*-values of both co-precipitation and mechanochemistry are largely less than one of the undoped K_2_FeO_4_, which implies that the doped cathodes are more stable than the undoped cathode.

### 3.5. Mechanism of the stability of the $SO_{4}^{2-}$-doped K_2_FeO_4_ cathodes

**Scheme 1.**
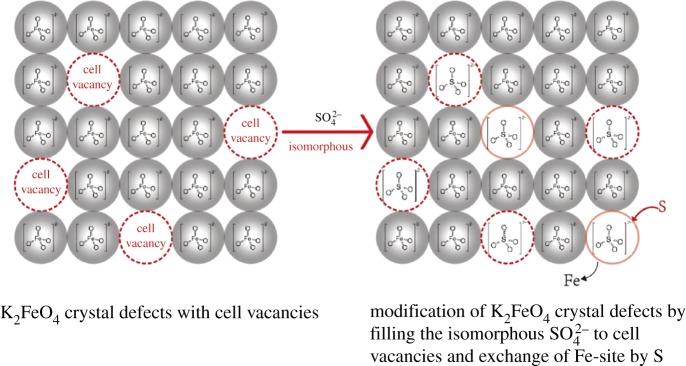
Mechanismic chemistry of the structural modification of isomorphous $SO_{4}^{2-}$-doped K_2_FeO_4_.

The above data exhibited a proper stability of the $SO_{4}^{2-}$-doped K_2_FeO_4_ cathodes. For understanding the stability, the proposed mechanismic chemistry is demonstrated in scheme 1.

Based on the analysis and data in the Introduction section, the $\mathrm{Fe}O_{4}^{2-}$ tetrahedron building block has a deformed structure with tension for basically showing a trend of an unstable crystal structure. Moreover, the crystal defect exists in the structure because of the cell vacancy or overfill, which dominates an instability of the crystalline. So, $SO_{4}^{2-}$ tetrahedron building block, an equivalent atom, unit cell and isomorphous crystalline with the $\mathrm{Fe}O_{4}^{2-}$ tetrahedral, can be orderly doped to the appropriate vacancies and replace $\mathrm{Fe}O_{4}^{2-}$ building block sites for filling the in/on-crystal defects and forming the isomorphous substitution of K_2_Fe_1−x_S_x_O_4_ complex salt. The reformed crystalline would be expected to be kept perfect. The alternative strategy was effective for improving the stability of K_2_FeO_4_ cathodes in this investigation.

## 4. Conclusion

Ferrates have been adapted to the cathode materials with the high energy, environmental benignity and low cost, which has been attracting a growing research attention. The inherent instability of $\mathrm{Fe}O_{4}^{2-}$ salts has restricted the advanced development of the alkaline super-iron battery. Based on the analysis of the instability from the structural defects and vacancies, the isomorphous $SO_{4}^{2-}$ was doped to K_2_FeO_4_ via a facile co-precipitation and mechanochemistry for the remediation of the discharge and stability of the battery. Summarily, the small amounts of K_2_SO_4_ were doped into the K_2_FeO_4_ in the calculated form of K_2_Fe_1−x_S_x_O_4_ by the isomorphous substitution. The doped K_2_FeO_4_ cathodes/batteries exhibited an excellent enhancement of the discharge capacity with an increase of about 10–30% compared to the undoped K_2_FeO_4_. Moreover, the stability of the K_2_FeO_4_ cathodes was obviously remediated by the isomorphous $SO_{4}^{2-}$ doping. The shelf time of the doped K_2_FeO_4_ cathodes was prolonged by increasing about 10% in comparison of the undoped K_2_FeO_4_ cathode. The two doping techniques had the same effect on the improvement of both discharge and stability. The desirable enhancements could be explained by the doping and reforming of the similar size and isomorphous $SO_{4}^{2-}$ building block to the $\mathrm{Fe}O_{4}^{2-}$ tetrahedral and crystalline for the isomorphous substitution and filling vacancies. This study enables a presentation of the experimental data to a stabilization of ferrates for use in the super-iron battery. Some investigations on the detailed structures and mechanisms are ongoing for fully understanding the two enhancements.

## Data accessibility

We have made all details available in our paper and uploaded the data to the Dryad Digital Repository: http://dx.doi.org/10.5061/dryad.mv789q6 [35].
